# Multiresistant-MRSA tricuspid valve infective endocarditis with ancient osteomyelitis locus

**DOI:** 10.1186/1471-2334-6-124

**Published:** 2006-07-26

**Authors:** Giuseppe Chesi, Andrea Colli, Carlos A Mestres, Gianpaolo Gambarati, Fabrizio Boni, Tiziano Gherli

**Affiliations:** 1 Department of Internal Medicine, "C. Magati" Hospital, Via Martiri della libertà 8, 42019 Scandiano (RE), Italy; 2 Department of Cardiac Surgery, University of Parma, Via Gramsci 13, 43100 Parma, Italy; 3 Department of Cardiovascular Surgery, Hospital Clinic, Villaroel 170, 08036 Barcelona, Spain

## Abstract

**Background:**

Methicillin-resistant *S. aureus *(MRSA) with low susceptibility to glycopeptides is uncommon.

**Case presentation:**

The case of a 50-year-old non-drug addict patient presenting with tricuspid valve infective endocarditis (IE) by MRSA resistant to vancomycin and linezolid is presented. There was response only to quinupristin/dalfopristin. He had a motorcycling accident four years before undergoing right above-the-knee amputation and orthopaedic fixation of the left limb. There were multiple episodes of left MRSA-osteomyelitis controlled after surgery and vancomycin therapy. MRSA isolated from the blood at the time of IE presented with the same profile than the isolated four years earlier. Sequential treatment with teicoplanin-cotrimoxazole and Linezolid associated to vancomycin – rifampicin – cotrimoxazole had no improvement. Infection was controlled after 28 days of therapy with quinupristin/dalfopristin.

**Conclusion:**

The literature presents only a few cases of MRSA IE not susceptible to glycopeptides in not drug addicted patients. This case shows the comparison of a highly-resistant MRSA after previous *S. aureus *osteomyelitis treated with glycopeptides. This is the first description of successful treatment of resistant-MRSA IE of the tricuspid valve complicated by multiple pulmonary septic infarction with quinupristin/dalfopristin

## Background

Infective endocarditis was an invariably fatal infection before the availability of antimicrobials. A significant percentage of patients still succumb to it despite aggressive treatment especially when infected with virulent organisms such as *Staphylococcus aureus*. Overall, approximately 20% of S. aureus isolates in Europe are reported as methicillin-resistant, whereas in US hospitals the prevalence ranges from 33% to 55%. The past few years have also witnessed an increase in life-threatening community-acquired infections caused by MRSA. Most infections with VISA (vancomycin-intermediate S. aureus) and VRSA (vancomycin-resistant S. aureus) have occurred in a setting of heavy prior use of glycopeptides and other antimicrobial agents. Emergence of reduced vancomycin susceptibility and clinical resistance in S. aureus increases the possibility that currently available antimicrobial agents may become ineffective for treating systemic infections.

## Case presentation

A 50 year-old non-insulin dependant diabetic male was admitted with the chief complaints of three-day fever and poor general status. He had a 3/6 diastolic murmur over the xyphoid process. Chest X-ray did not show abnormalities. Abdominal ultrasound showed splenomegaly. His past history included the following: In June 2000 he had a road traffic accident resulting in multiple trauma, requiring right above-the-knee amputation and orthopaedic fixation of the left lower limb. He later presented with multiple episodes of osteomyelitis of this limb treated with iv. antibiotics. Prosthetic shafts were removed in June 2002 and MRSA isolated. Three months later he presented with a spontaneous fracture of the limb and required new surgical fixation due to infectious pseudoarthrosis. MRSA was isolated again. In October 2003 he was re-operated and underwent auto-transplantation of corticospongiosa. During these episodes he was given teicoplanin, cotrimoxazole, rifampicin, tetracycline and quinolones at intervals of 7 to 20 days at another institution in a distant site. Table 1 shows the summary of the multiple events.

**Table 1:** Summary of multiple events

**Period**	**Hospital Admission**	**Cause**	**Isolated pathogen**	**Antibiotic therapy**
**June 2000**	ICU	Road Traffic accident	-	-
**August 2000 – December 2000**	Rehabilitation Unit	Rehabilitation, Orthopaedic fixation, osteomyelitis left leg	-	Ciprofloxacin 500 mg/12 h/21 days Amoxicillin/clavulanic acid 1 g/12 h/28 days
**June 2002**	Orthopaedic Unit	Re-Orthopaedic fixation	MRSA	Minocycline 100 mg/12 h/14 days Cotrimoxazole 480 mg./12 h/8 days Teicoplanin 200 mg/24 h/8 days
**September 2002**	Orthopaedic Unit	Spontaneous limb fracture – Orthopaedic fixation	MRSA	Teicoplanin 400 mg/24 h/8 days Rifampicin 600 mg/12 h/10 days
**February 2003 – May 2003**	Rehabilitation Unit	Rehabilitation	-	-
**October 2003**	Orthopaedic Unit	Autotransplantation of corticospongiosa	MRSA	Ciprofloxacin 500/12 h/14 days Teicoplanin 200 mg/24 h/8 days
**June 2004 – August 2004**	Internal Medicine Unit and Cardiac Surgery Unit	Tricuspid valve endocarditis	MRSA	Cefixime 800 mg/2 days Oxacillin 16 g/24 h/3 days + Gentamycin 240 mg/24 h/3 days Teicoplanin 1200 mg/24 h/8 days + Cotrimoxazole 960 mg/24 h/8 days Linezolid 600 mg/24 h/7 days Vancomycin 3 g/24 h/12 days + Rifampicin 600 mg/24 h/12 days+ Cotrimoxazole 1440 mg/24 h/12 days Quinupristin/dalfopristin 750 mg/8 h/36 days

**Figure 1:**
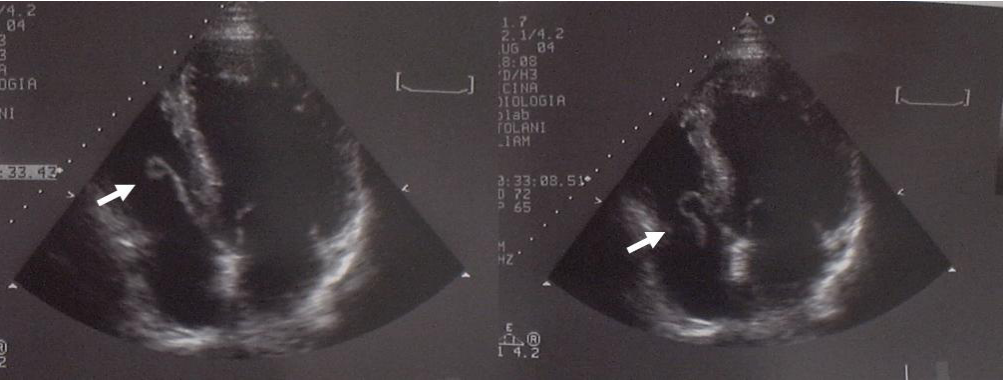
Transthoracic echocardiogram showing a vegetation on the septal leaflet of the tricuspid valve.

**Figure 2:**
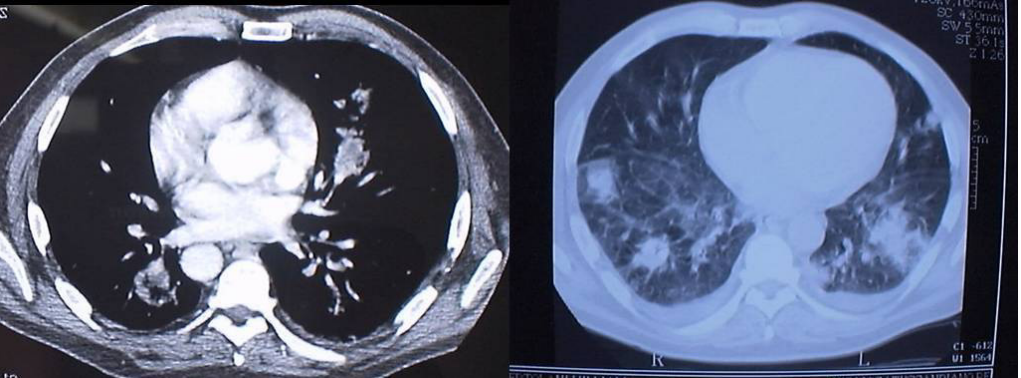
CT scan showing multiple areas of pulmonary infarction.

In June 2004 he was admitted to our institution with the above mentioned complaints. Transthoracic echocardiography (TTE) showed a large vegetation (size 2.5 × 0.8 cm) on the anterior leaflet of the tricuspid valve (Figure 1) and severe valve insufficiency, with a normal left ventricular ejection fraction. Transesophageal echocardiography (TEE) disclosed other pathologic findings. A total body computed tomography scan was performed showing multiple bilateral areas suggesting pulmonary infarction (Figure 2). A labelled leucocyte scintigraphy didn't reveal any specific infective localization.

The patient was treated with an association of oxacillin 16 gr/day and gentamycin 240 mg/day that was shifted two days later to teicoplanin (1200 mg/12 h/IV) for the first 3 days followed with 1200 mg/day/IV) and cotrimoxazole (480 mg/12 h/IV) as MRSA was isolated. The MRSA was resistant to clindamycin (MIC>8 mcg/ml), erythromycin (MIC>8 mcg/ml), gentamycin (MIC>16 mcg/ml), norfloxacin (MIC>16 mcg/ml), ofloxacin (MIC>8 mcg/ml), oxacillin (MIC>4 mcg/ml), penicillin (MIC>0.8 mcg/ml), and tobramycin (MIC>16 mcg/ml). The MRSA resulted susceptible to rifampicin (MIC<0.5 mcg/ml), teicoplanin (MIC<0.5 mcg/ml), tetracicline (MIC<1 mcg/ml), cotrimoxazole (MIC<10 mcg/ml) and vancomycin (MIC<1 mcg/ml). Linezolid 600 mg daily iv was started 8 days later because of worsening condition (Linezolid was not tested in-vitro). However this proved to be ineffective as the patient still presented fever and positive blood cultures for MRSA maintaining the same strains. A new TTE was performed showing the presence of a new vegetation on the anterior leaflet of the tricuspid valve.

Linezolid was then replaced with vancomycin (500 mg/6 h/IV), rifampicin (600 mg/8 h/IV) and cotrimoxazole (480 mg/8 h/IV) that was again ineffective and after 12 days of therapy was suspended. After 27 days of ineffective therapy the patient condition was very poor; TTE showed new vegetations on the posterior leaflet of the tricuspid valve with severe insufficiency and large pulmonary infarctions. Quinupristin/dalfopristin were at 7.5 mg-Kg/8 h/IV. After three days of therapy the patient became afebrile and his condition improved. After 25 days of therapy with quinupristin/dalfopristin, biological markers for infection became normal and the patient underwent left above-the-knee amputation as prevention treatment for new infections. The therapy with quinupristin/dalfopristin was suspended after 42 days. A Transesophageal echocardiography and a thoracic computed tomography scan were performed at the end of antibiotic therapy and at 18 months after discharge showed complete regression of the tricuspid vegetation, only mild valve insufficiency and absence of residual pulmonary infarction.

Infective endocarditis is an uncommon disease. A recent publication has shown that the incidence of IE is about 31 cases per million inhabitants. Right-sided IE represents 10% of all cases with tricuspid valve involvement in 90% of cases. Moss and Munt showed the right-sided IE is more frequent in drug-addicts (63%) [2,3]. We present a case of tricuspid IE complicated by multiple pulmonary septic infarctions originated probably from a focus of chronic osteomyelitis. The interesting aspect of this case is not only the acute clinic presentation but also the high grade of clinical resistance to the different antibiotic regimens used [4]. The patient did not respond to the use of glycopeptides, teicoplanin and vancomycin always in association with other drugs despite the positive in vitro susceptibility to these antibiotics [5]. The use of linezolid [6], a wide spectrum antibiotic, with a very good reported action on the lungs [7] resulted ineffective with a concomitant dramatic deterioration of the clinical condition of the patient. The use of quinupristin/dalfopristin [8] as salvage therapy resulted in a very quick improvement after three days and resulted in complete cure of tricuspid valve IE and septic embolic lung abscesses. The antibiotic therapy was continued for four weeks as recommended by the American Heart Association guidelines [9] for complicated right-sided endocarditis diminishing the rate of relapses due. The decision to perform a left above-the-knee amputation was considered as the only possible guarantee of removal of chronic osteomyelitis focus and was performed with the patient on quinupristin/dalfopristin antibiotic treatment.

## Conclusion

In this paper, we firstly reported a non-operated patient with tricuspid valve endocarditis by *MRSA *complicated by multiple pulmonary septic infarction cured with quinupristin/dalfopristin. The use of these antibiotic agents opens new perspectives in the management of severely ill patients with invasive valve endocarditis infection.

## Abbreviations

MRSA: Methicillin-resistant *S. aureus*

IE: infective endocarditis

TTE: Transthoracic echocardiography

TEE: Transesophageal echocardiography

MIC: minimal inhibitory concentration

## Competing interests

The author(s) declare that they have no competing interests.

## Authors' contributions

AC, GC, were involved in conception, design and drafting. CAM and TG made a critical revision of the manuscript. FB, GG participated in the writing of the first draft. All authors improved the manuscript and approved its final version.

## Pre-publication history

The pre-publication history for this paper can be accessed here:


